# Supplementary material for: In situ coherent diffractive imaging
Source: Nat Commun. 2018 May 8;9:1826. doi: 10.1038/s41467-018-04259-9 (PMC5940918; doi:10.1038/s41467-018-04259-9)
Supplement: Supplementary file 1 — Supplementary Information [file 41467_2018_4259_MOESM1_ESM.pdf]

# In situ coherent diffractive imaging

Supplementary Information

Lo et al.

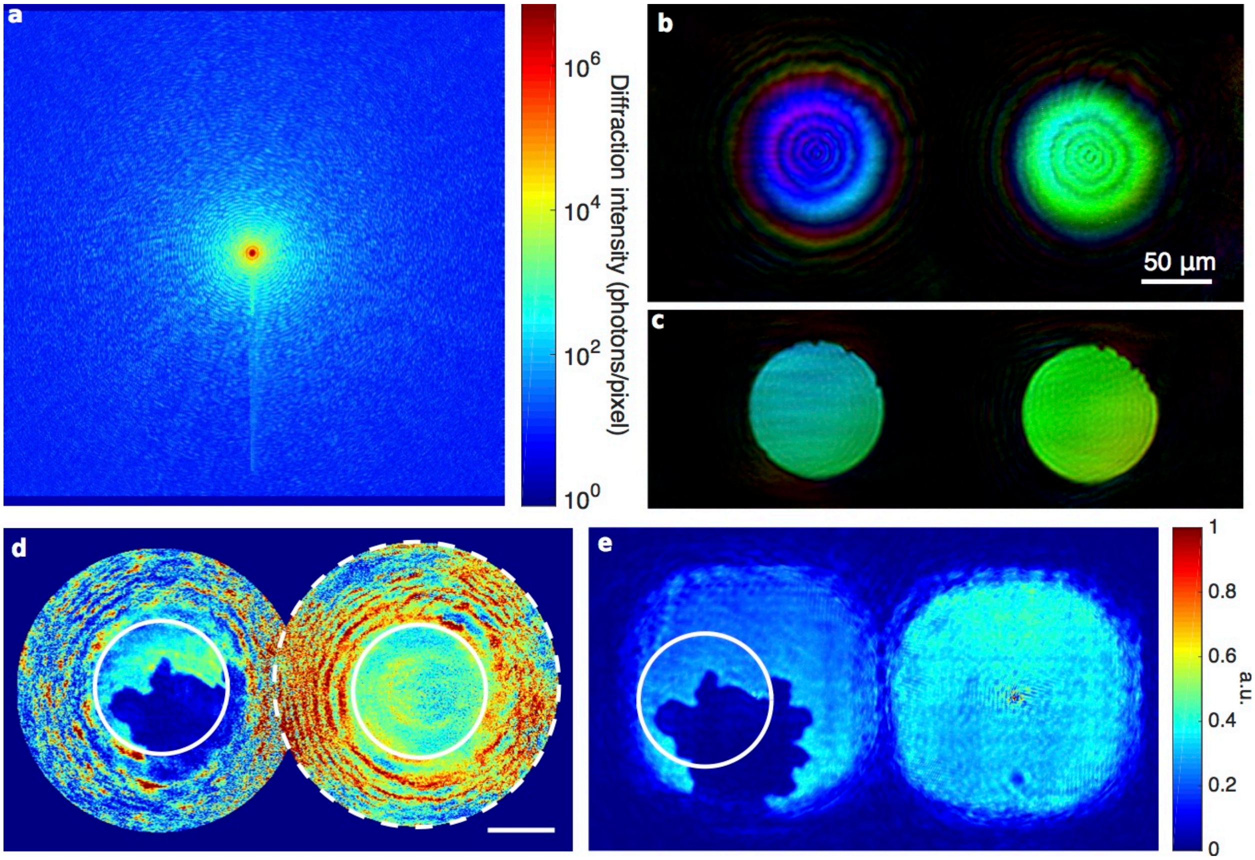


**Supplementary Figure 1 | Optical laser data and reconstructions.** (**a**) Representative diffraction pattern collected in the Pb dendrite growth experiment. (**b**) The complex illumination function on the sample plane, recovered from ptychography and used for the in situ CDI reconstruction. Brightness and hue represent magnitude and phase, respectively. (**c**) The illumination function propagated (460 µm) onto the aperture plane. (**d**) In situ CDI reconstruction of the 0.9 V frame in the Pb dendrite growth process, showing the fully reconstructed field of view. The two solid white circle represent the illumination field at the pinhole plane, the left solid white circle indicates the region used for Fig. 3a, and dashed white circle denotes the region masked for the static structure constraint. Note a loose support was used on the illumination function to bound the aperture area. Scale bar 50 µm. **e**, Ptychographic reconstruction of the same voltage frame as (**d**). Solid white circle indicates the region used for Fig. 3b.

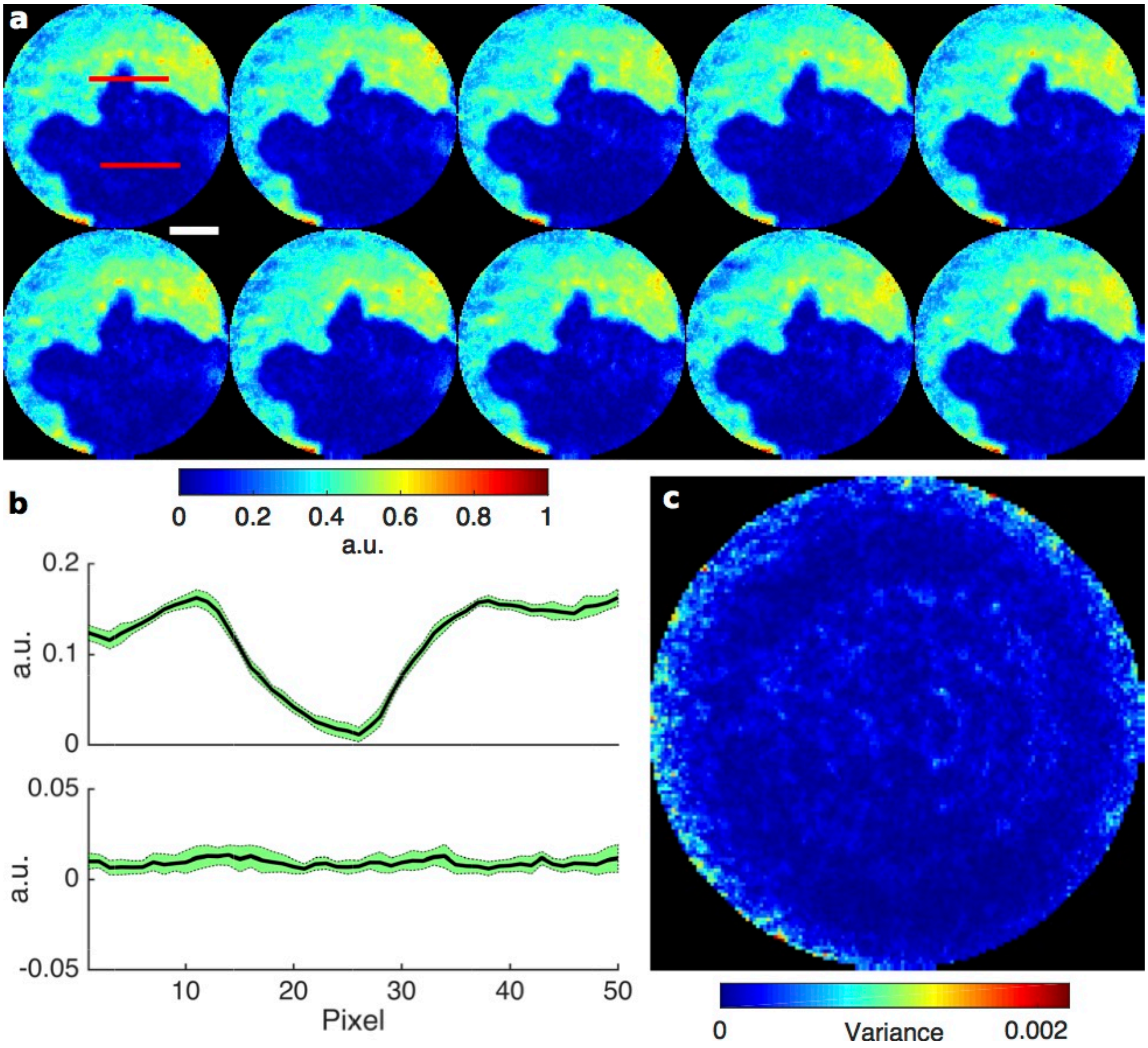


**Supplementary Figure 2 | In situ CDI reconstruction consistency.** (**a**) Ten independent runs of the in situ CDI reconstruction of the Pb dendrite growth process with the applied voltage of 0.9 V. Each run used a random dynamic and static function as an initial input. Scale bar, 20 μm. (**b**) Average line-scans across two regions of the reconstructions (red lines in (**a**)) with the standard deviation in green. (**c**) the variance map of the reconstructions, showing good consistency of the ten independent runs.

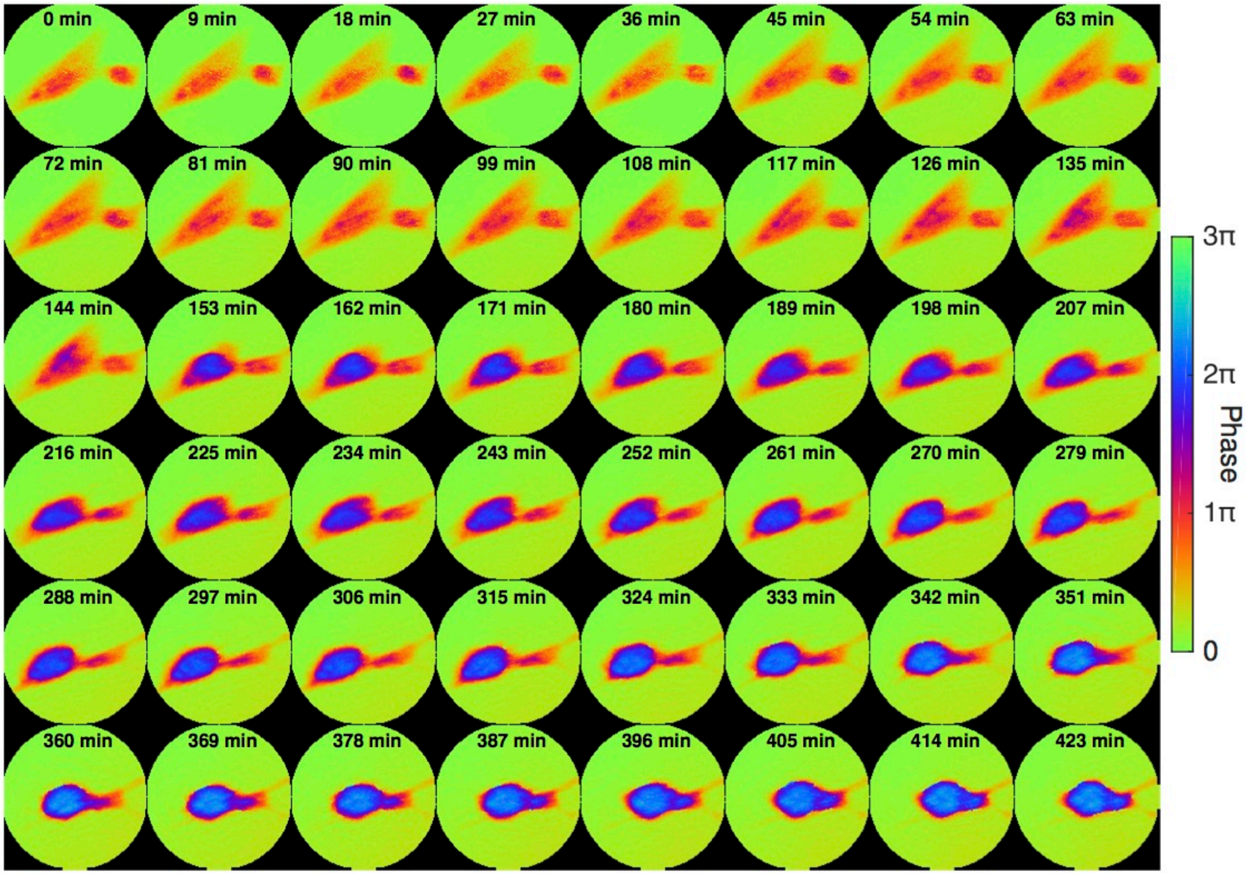


**Supplementary Figure 3 | Cell fusion dynamics result from ptychography.** Phase images of the fusion of glioblastoma cells reconstructed by ptychography. Each frame corresponds to the same region as the in situ CDI reconstruction in Fig. 4. Internal cellular structures reconstructed using both methods show good agreement, validating in situ CDI's ability to simultaneously reconstruct a time series of diffraction patterns.

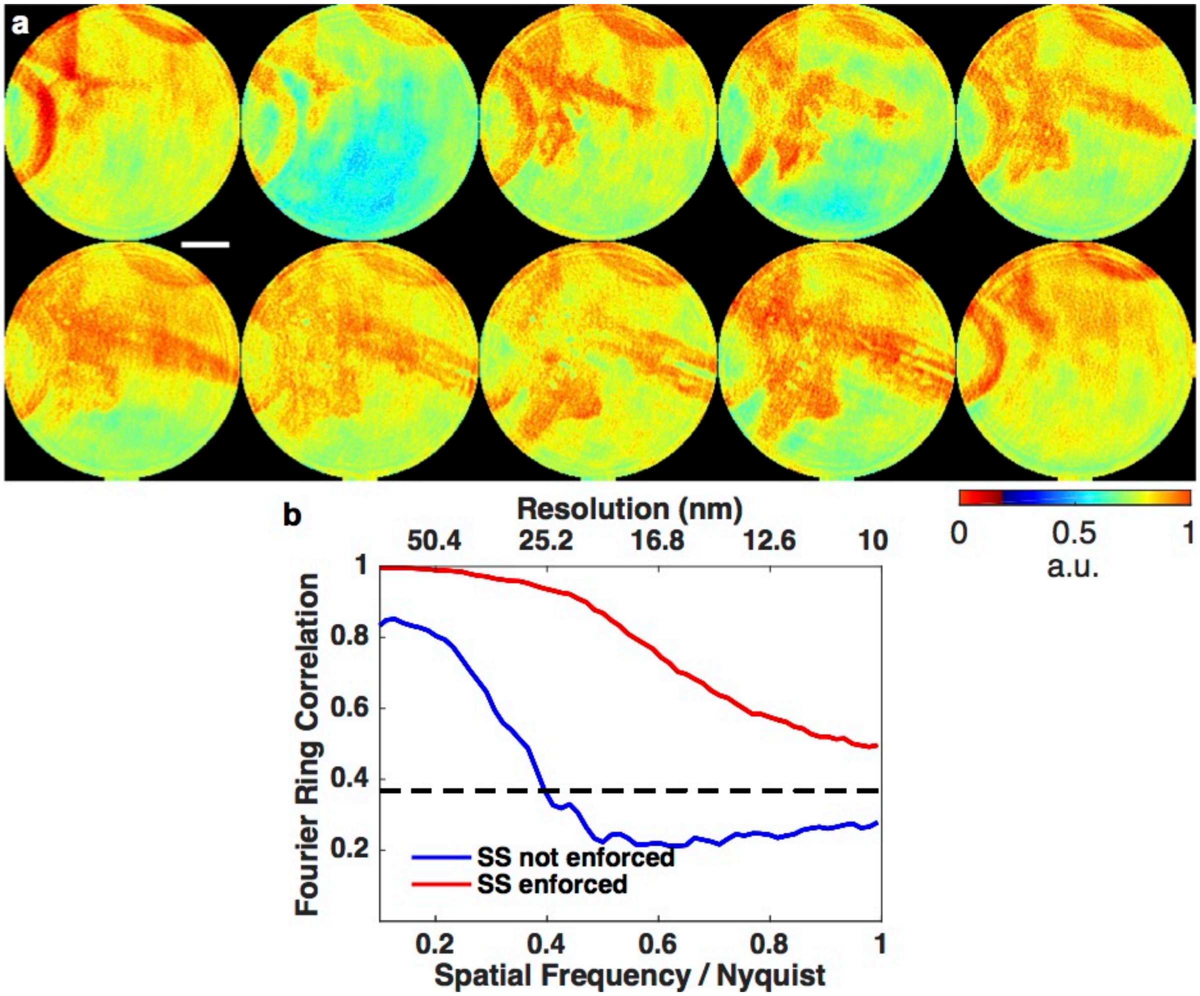


**Supplementary Figure 4 | Results without static constraint enforcement.** (**a**) In situ CDI reconstructions without enforcing the static structure (SS) constraint, exhibiting poor reconstruction quality than that with the SS constraint enforced. Scale bar, 200 nm. (**b**) Fourier ring correlation indicates that enforcing the SS constraint can improve the resolution of the reconstructions.

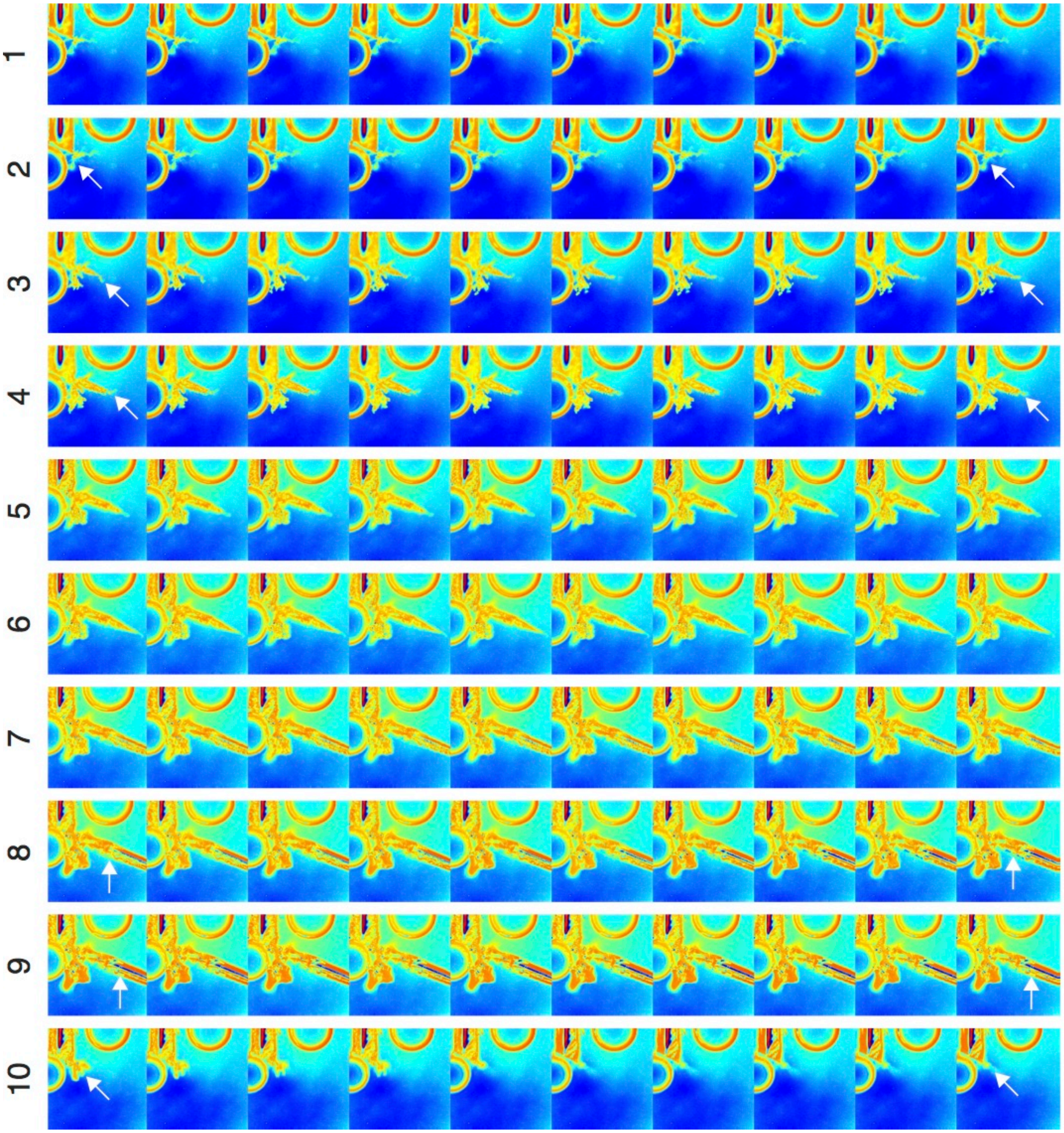


**Supplementary Figure 5 | All frames used for constructing the Pb dendrite dynamic structure model**. Each row represents 10 1-ms images used to calculate a diffraction pattern with a temporal resolution of 10 ms. The arrows indicate fine feature changes of the images.
