# Supplementary material for: In situ coherent diffractive imaging
Source: Nat Commun. 2018 May 8;9:1826. doi: 10.1038/s41467-018-04259-9 (PMC5940918; doi:10.1038/s41467-018-04259-9)
Supplement: Supplementary file 3 — Description of Additional Supplementary Files [file 41467_2018_4259_MOESM3_ESM.pdf]

Description of Additional Supplementary Files:

Supplementary Movie 1. **In situ CDI experiment with a materials science sample.** The magnitude of the complex exit waves reconstructed by in situ CDI, capturing the growth of Pb dendrites on Pt electrodes immersed in an aqueous solution of $Pb(NO_3)_2$ as a function of the applied voltage.

Supplementary Movie 2. **In situ CDI experiment with a biological sample.** Phase images of the fusion of glioblastoma cells reconstructed by in situ CDI.
